# Proof-of-Concept Gene Editing for the Murine Model of Inducible Arginase-1 Deficiency

**DOI:** 10.1038/s41598-017-02927-2

**Published:** 2017-05-31

**Authors:** Yuan Yan Sin, Phillipe R. Price, Laurel L. Ballantyne, Colin D. Funk

**Affiliations:** Department of Biomedical and Molecular Sciences, Queen’s University, Kingston, Ontario Canada

## Abstract

Arginase-1 deficiency in humans is a rare genetic disorder of metabolism resulting from a loss of arginase-1, leading to impaired ureagenesis, hyperargininemia and neurological deficits. Previously, we generated a tamoxifen-inducible arginase-1 deficient mouse model harboring a deletion of *Arg1* exons 7 and 8 that leads to similar biochemical defects, along with a wasting phenotype and death within two weeks. Here, we report a strategy utilizing the Clustered, Regularly Interspaced, Short Palindromic Repeats (CRISPR)/CRISPR-associated protein 9 (Cas9) system in conjunction with *piggyBac* technology to target and reincorporate exons 7 and 8 at the specific *Arg1* locus in attempts to restore the function of arginase-1 in induced pluripotent stem cell (iPSC)-derived hepatocyte-like cells (iHLCs) and macrophages *in vitro*. While successful gene targeted repair was achieved, minimal urea cycle function was observed in the targeted iHLCs compared to adult hepatocytes likely due to inadequate maturation of the cells. On the other hand, iPSC-derived macrophages expressed substantial amounts of “repaired” arginase. Our studies provide proof-of-concept for gene-editing at the *Arg1* locus and highlight the challenges that lie ahead to restore sufficient liver-based urea cycle function in patients with urea cycle disorders.

## Introduction

Arginase-1 (ARG1), highly expressed in liver, is the final enzyme of the urea cycle. Inherited deficiency of this enzyme results in hyperargininema with progressive neurological and intellectual impairment, growth retardation and occasional episodes of hyperammonemia. Treatment of arginase-1 deficiency consists of supportive measures including pharmacologic agents to remove excess nitrogen and a low-protein diet. No cure is available^1, 2^, although liver transplantation restores liver arginase-1 and appears to prevent progression of the neurological symptoms^3^. We and others have previously generated and characterized similar tamoxifen-inducible mouse models of arginase-1 deficiency via Cre-mediated excision of *Arg1* exons 7 and 8 that lead to mice nearly devoid of hepatic Arg1 enzyme activity with a severe wasting phenotype and death approximately two weeks later^4–6^. Strategies to rescue the lethal phenotype in mice including ornithine supplementation, low-protein diet, nitrogen scavengers and a transgenic approach were unsuccessful. Gene therapeutic approaches have also been implemented as a means to correct the lethal phenotype of inducible and global Arg1 knockout mouse models using adenoviral and adeno-associated viral (AAV) vectors expressing Arg1. However, none have rescued every feature of the genetic deficiency^7–12^.

Induced pluripotent stem cells (iPSCs) have the capability to undergo unlimited self-renewal and differentiate into all cell types^13–17^, thereby holding great potential for stem cell-based therapies for many untreatable diseases, including urea cycle disorders. Concurrent with the application of iPSC technology, the emerging Clustered, Regularly Interspaced, Short Palindromic Repeats (CRISPR) CRISPR-associated protein 9 (Cas9) genome editing technology^18–22^ has garnered great interest for potential therapeutic applications due to its ease of use, efficacy, specificity and versatility. It is composed of a DNA-cutting enzyme Cas9 and a guide RNA (gRNA) containing 20 nucleotides of identity to a target sequence proximal to a protospacer-adjacent motif (PAM). Cas9 is directed to the intended target site by a gRNA to induce double-stranded breaks (DSBs), which are subsequently repaired through endogenous DNA repair mechanisms, either by error prone non-homologous end joining (NHEJ) or homology-directed repair (HDR) for precise genome alterations in the presence of exogenously introduced homologous DNA template.

Here, we use iPSCs derived from our inducible model in combination with CRISPR/Cas9 and *piggyBac* transposon^23, 24^ systems to provide proof-of-concept for biallelic targeted gene repair of multi-exon deletion *in vitro* in the parental cells, iPSC-derived hepatocyte-like cells (iHLCs) and cells differentiated to macrophages.

## Results

### Generation and characterization of Arg1-deficient (*Arg1*^*Δ*^) iPSCs

**Figure 1:**
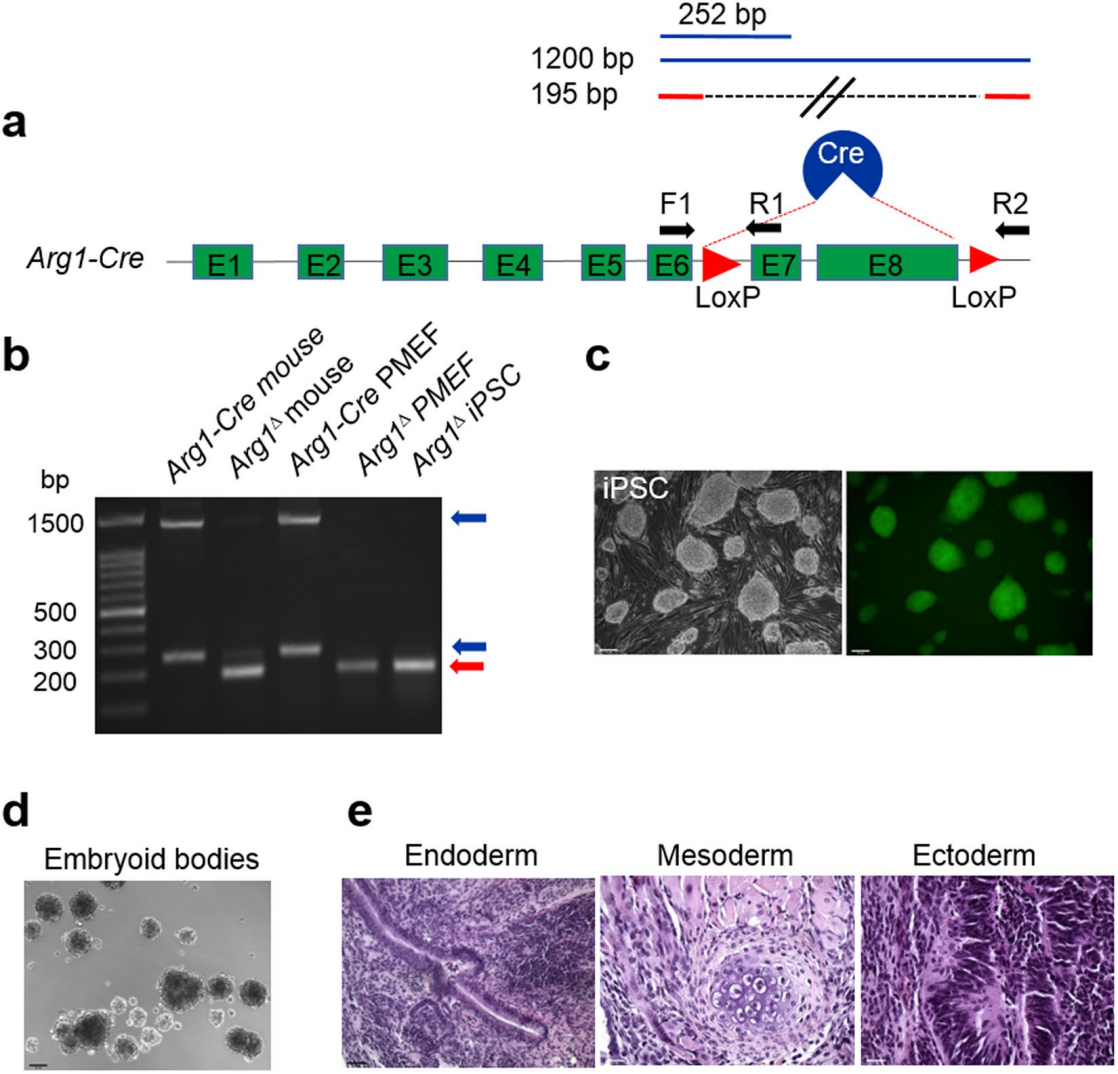
Generation of Arg1-deficient iPSCs from PMEF cultures. (**a**) Schematic diagram depicting the genotype of cell source. The arrows indicate the positions of the primers used for genotyping the resulting cells. (**b**) PCR genotyping to confirm the deletion of exons 7 and 8. *Arg1*-*Cre PMEFs* showed bands at 1.2 kb and 252 bp (indicative of intact exons 7 and 8), while both *Arg1* ^*∆*^ PMEFs and derived iPSCs produced only one band at 195 bp. Skin fibroblasts isolated from a tamoxifen-induced Arg1-deficient mouse were used as a positive control, while untreated Arg1-Cre PMEFs served as negative control. (**c**) Alkaline phosphatase as a pluripotent marker. (**d**) Formation of embryoid bodies and (**e**) teratoma assay to demonstrate trilineage differentiation *in vitro* and *in vivo*, respectively. Shown are gutlike epithelial tissues, cartilage and neural tissues, depicting the formation of endoderm, mesoderm and ectoderm, respectively.

**Figure 2:**
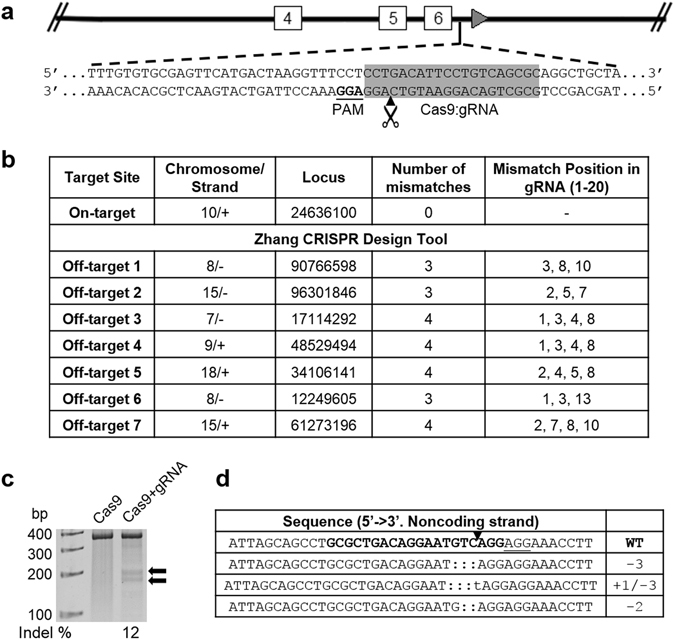
CRISPR/Cas9-mediated gene targeting in mouse iPSCs. (**a**) Schematic diagram showing the site of *Arg1* gene modification using CRISPR/Cas9. The gRNA was designed to target a 20 nt region in intron 6 of *Arg1*. (**b**) Cas9:gRNA off-target analysis. A table showing the output from CRISPR design tool. The on-site target is given at the top, followed by off-target sequences organized in decreasing Cas9-cleavage probability. (**c**) Detection of Cas9:gRNA-mediated on-target cleavage of *Arg1* by the Surveyor nuclease assay. The cleavage products were shown as extra bands (149 bp + 197 bp) indicated by the arrows. Mutation frequency (indel) was calculated by measuring the band intensities. (**d**) Sequencing data from TOPO-cloned PCR amplicons of CRISPR/Cas9-modified genomic DNA showing a few examples of NHEJ-mediated indel mutations at the desired location. gRNA target site is in bold. Deletions are shown in dashes. Nucleotide substitution is shown in lowercase. The net change in length caused by each indel mutation is to the right of each sequence.

**Figure 3:**
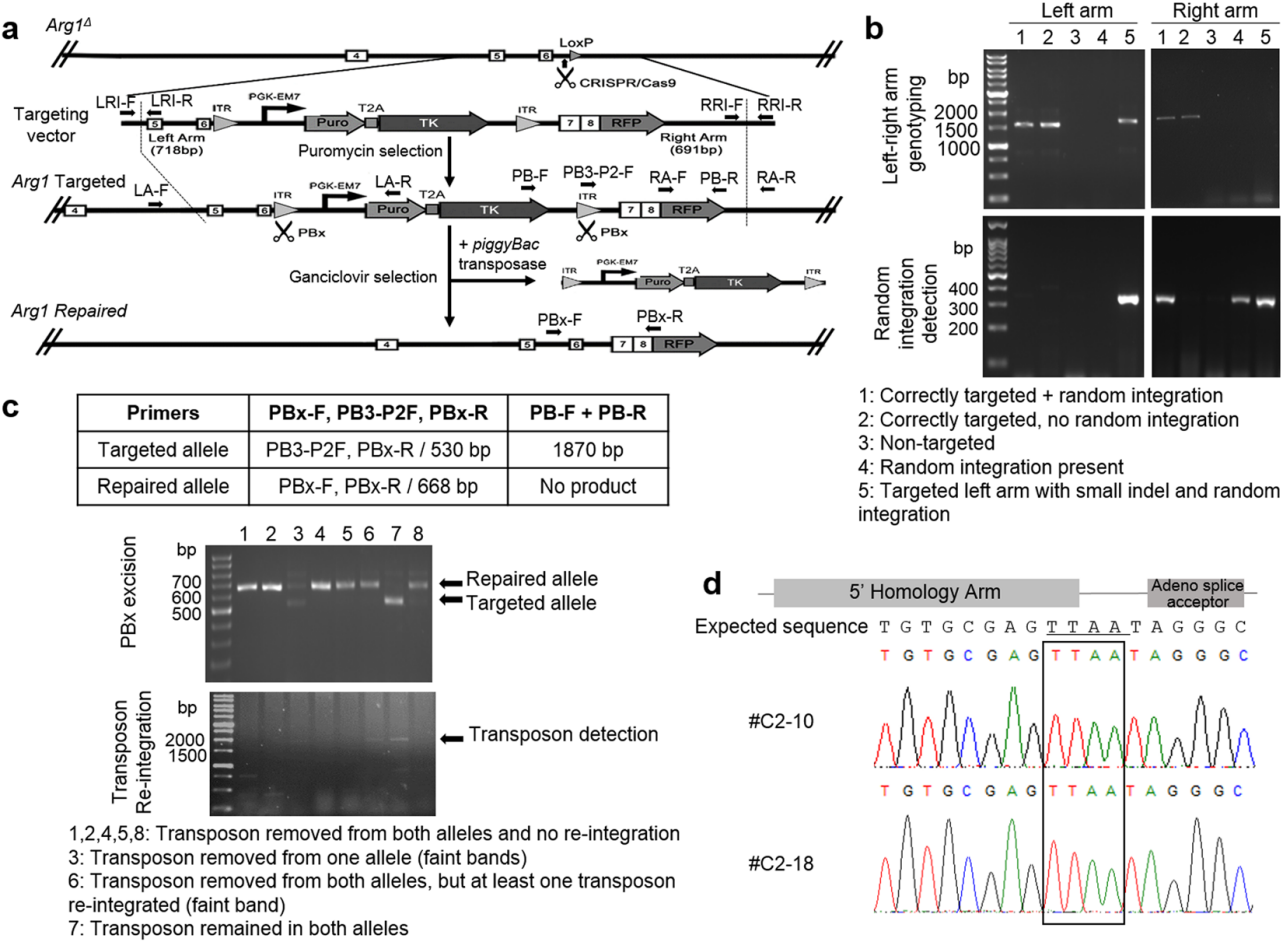
Reincorporation of *Arg1* exons 7 and 8 via homology-directed repair. (**a**) The experimental strategy for precise genome modification using CRISPR/Cas9 and *piggyBac* transposon methodology. Arrows indicate primers for PCR-based screening to confirm the selected clones. The remnant LoxP left from the initial Cre-excision of exons 7 and 8 would be removed upon targeting vector integration. PBx, *piggyBac* transposase; T2A, viral sequence for ribosomal skipping; Puro, puromycin; ITR, inverted terminal repeat; TK, thymidine kinase. (**b**) Representative gel images of puromycin-resistant iPSC colonies screened for correctly targeted clones. Each homology arm was amplified independently by PCR. Amplicon sizes of the left and right arm were 1530 and 1660 bp, respectively, while random integration should yield a product of ≈300 bp. (**c**) PCR-based genotyping of alleles after *piggyBac* excision. Upper panel shows primer combination to uniquely identify different alleles. Lower panel shows a representative gel of different banding patterns and corresponding genotypes. Amplicon sizes of the repaired and targeted alleles were 668 bp and 530 bp, respectively. (**d**) Footprint analysis by sequencing. Sequences in transposon-free repair clones indicating the exact repair after transposon excision. TTAA target sites are boxed.

An iPSC-based strategy was devised to gene edit the murine model of inducible Arg1 deficiency (Figs 1–3). Primary mouse embryo fibroblasts (PMEFs) isolated from Arg1-Cre mouse embryos (Fig. 1a) were treated with 4-hydroxy-tamoxifen (4-OHT) *in vitro* for the excision of floxed exons 7 and 8 to inactivate *Arg1*. DNA from *Arg1*-*Cre* mice yield 1.2 kb and 252 bp bands, indicative of intact exons 7 and 8. Successful excision of exons 7 and 8 of *Arg1* in PMEFs and derived cells yields a band of 195 bp characteristic of the *Arg1* ^*Δ*^ allele (Fig. 1b). Arg1-deficient iPSCs were generated using lentiviral transduction of re-programming factors Klf4, Oct4, Sox2 and c-Myc^25^. Colonies exhibited typical embryonic stem cell-like morphology: dome-shaped, refractile densely packed colonies with high nuclear to cytoplasmic ratios, prominent nuclei and distinct colony border after transduction. The undifferentiated state showed alkaline phosphatase staining (Fig. 1c), along with the ability to form embryoid bodies under appropriate culture conditions (Fig. 1d). Moreover, teratoma formation was observed in immune-deficient mice, showing differentiation capability in forming derivatives of all three germ layers as depicted in Fig. 1e.

### CRISPR/Cas9-mediated reincorporation of deleted exons via homology-directed repair

An optimal gRNA target candidate was chosen based on the highest scoring for on-target and sequence conservation with minimal potential off-target effects using an *in silico* prediction tool. This gRNA was designed *de novo* to target intron 6 of the *Arg1* locus for the reincorporation of exons 7 and 8 (Fig. 2a). By *in silico* analysis, we found seven hypothetical off-target sites for this gRNA, which all had at least three mismatched bases (Fig. 2b). Hsu *et al*.^26^ demonstrated that Cas9 showed no measurable cleavage in targets that had greater than two mismatches, independent of their location within the genome. Surveyor nuclease cleavage at the mismatches produced products of 149 bp and 197 bp (Fig. 2c). This heteroduplex assay determined the ability of gRNA-guided Cas9 to induce DSBs in a precise and predictable manner, with mutation frequencies up to 12% in mouse iPSCs. Sequencing results confirmed cleavage in the target region as evidenced by the presence of NHEJ-induced indels (Fig. 2d).

Once measurable cutting efficiency was achieved, we performed HDR by introducing a donor targeting vector together with Cas9:gRNA into Arg1-deficient mouse iPSCs via electroporation. The targeting vector consists of exons 7 and 8 cDNA fused to an RFP monomeric-encoding segment with *piggyBac* inverted terminal repeats (ITR) flanking a *PGK*-*puro*-*tk* cassette along with two flanking homology arms of the target region to facilitate homologous recombination (Fig. 3a). An HDR stimulatory compound, L-755,507 was added to enhance CRISPR/Cas9-mediated HDR efficiency^27^. Puromycin-resistant mouse iPSC colonies obtained after electroporation were screened for proper targeting by PCR (Fig. 3b) and genotype confirmed by sequencing (data not shown). 5/23 iPSC clones were identified as targeted with no random integration, 10/23 were targeted correctly only on one arm and 8/23 were non-targeted. Two correctly targeted clones (clones #C2 and #C15) were chosen for subsequent *piggyBac* excision experiments. *piggyBac* transposase electroporation, followed by ganciclovir selection yielded 20 drug-resistant colonies from the #C2 line, 7 which were transposon-free clones, showing a single band at 668 bp indicating biallelic excision (Fig. 3c). Molecular genotypes can be inferred from the different banding patterns observed. Sequence analysis showed correct in-frame integration and the presence of the characteristic “footprint” TTAA sequence^28^ at the site of transposase excision (Fig. 3d). Additionally, 9/20 clones showed transposon removal from both alleles with at least one transposon re-integrated. 4/20 clones were heterozygous. In addition, 11 ganciclovir-resistant colonies were obtained from the #C15 line, all heterozygous.

### Hepatic differentiation of mouse iPSCs

**Figure 4:**
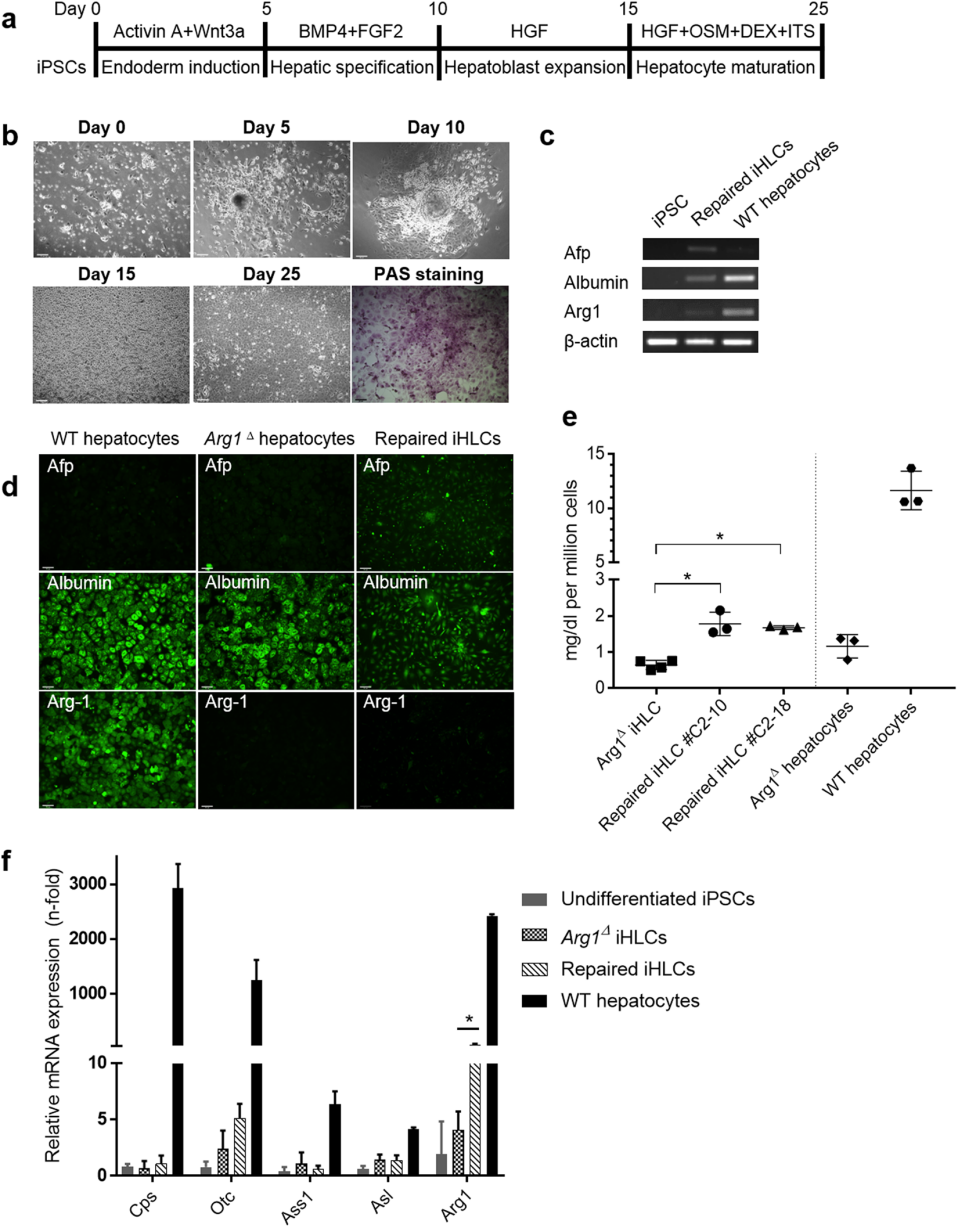
Generation of hepatocyte-like cells from repaired mouse iPSCs. (**a**) Schematic diagram showing the stepwise hepatic differentiation protocol. (**b**) Representative phase contrast images showing sequential morphological changes in iPSCs at different stages of differentiation (10x magnification). The cells are positive for PAS staining at day 25 after the start of the differentiation procedure. (**c**) RT-PCR gene expression of iHLCs for the hepatocyte markers alpha-fetoprotein (Afp), albumin and Arg1. Expression of genes was normalized to β-actin. (**d**) Representative immunostaining images from two independent batches of hepatic differentiation showing the expression profile. (**e**) Functional characterization of iHLCs. After 24 h of culture, supernatants were collected for urea production assay. Values were normalized by the number of cells seeded. Values are mean ± SD for n = 3 − 4. (**f**) qPCR expression analysis of the five urea cycle enzymes. The C_t_ values of all genes were normalized to the C_t_ values of 18S rRNA. The y-axis represents the fold-change of gene expression compared with undifferentiated iPSCs. Values are mean ± SD for n = 3. Statistical significance between groups was determined by Student’s *t*-test (*P < 0.05).

Next, we sought to examine whether *Arg1* gene editing resulted in functional correction in the repaired cells. Since *Arg1* is not expressed to any significant extent in iPSCs, but, rather, in highest amounts in hepatocytes, the repaired clones (#C2-10 and #C2-18) and parental non-edited cells were subjected to hepatic differentiation using a stepwise protocol, which consists of definitive endoderm induction, hepatic specification, hepatoblast formation and hepatocyte maturation (Fig. 4a). At the end of this 25-day differentiation protocol, iPSC-derived hepatocyte-like cells (named iHLCs hereafter) exhibited characteristic hepatocyte morphology such as cobblestone appearance, distinctive round nuclei and well-demarcated cell-cell borders with glycogen storage function confirmed by Periodic acid-Schiff (PAS) staining (Fig. 4b). RT-PCR (Fig. 4c) and immunohistochemical (Fig. 4d) analysis showed expression of the hepatic marker albumin (Alb), as well as alpha-fetoprotein (Afp) in iHLCs but only the former in mature hepatocytes isolated from C57BL/6 mice indicating that the iHLCs retain an immature hepatocyte phenotype. A faint band indicating weak expression of *Arg1* in iHLCs, compared to WT hepatocytes was present. The level of urea production was higher in both repaired iHLC lines compared to their non-repaired counterpart; however, the level was still considerably low compared to WT hepatocytes (Fig. 4e). The minimal ureagenesis observed in the *Arg1* ^*Δ*^ hepatocytes is likely residual expression resulting from incomplete tamoxifen-mediated removal of exons 7 and 8. Nevertheless, our observations are in accordance with a previous report that showed consistent expression of *Afp* accompanied by low *Arg1* expression and urea synthesis at the final stage of differentiation relative to primary hepatocytes^29^. To evaluate the expression of other urea cycle enzymes, we performed quantitative real-time PCR (qRT-PCR) and observed scarce expression of the five main ureagenic enzymes, providing further evidence of incomplete maturation (Fig. 4f). Although repaired iHLCs showed significant higher *Arg1* expression when compared to their iPSC and non-repaired counterparts, the level is still distinctly lower than in normal adult hepatocytes. Additionally, our strategy was designed to distinguish endogenous Arg1 expression from gene edited Arg1 repair and to discriminate signal above cellular autofluorescence by incorporation of a monomeric form of RFP^30^ fused to the carboxy terminus of the enzyme. Previously, we had demonstrated that Arg1 is fully functional with an eGFP tag placed in the same location^11, 12^. However, fluorescent imaging of the gene-edited iHLCs did not demonstrate red fluorescence but only very weak green fluorescence (Fig. 4d). These data suggested that either the cells were not mature enough for high-level Arg1 expression by the chosen differentiation protocol^29^ and/or that there were problems with maturation of the RFP fluorophore (see Discussion).

### Differentiation of mouse iPSCs to macrophages

**Figure 5:**
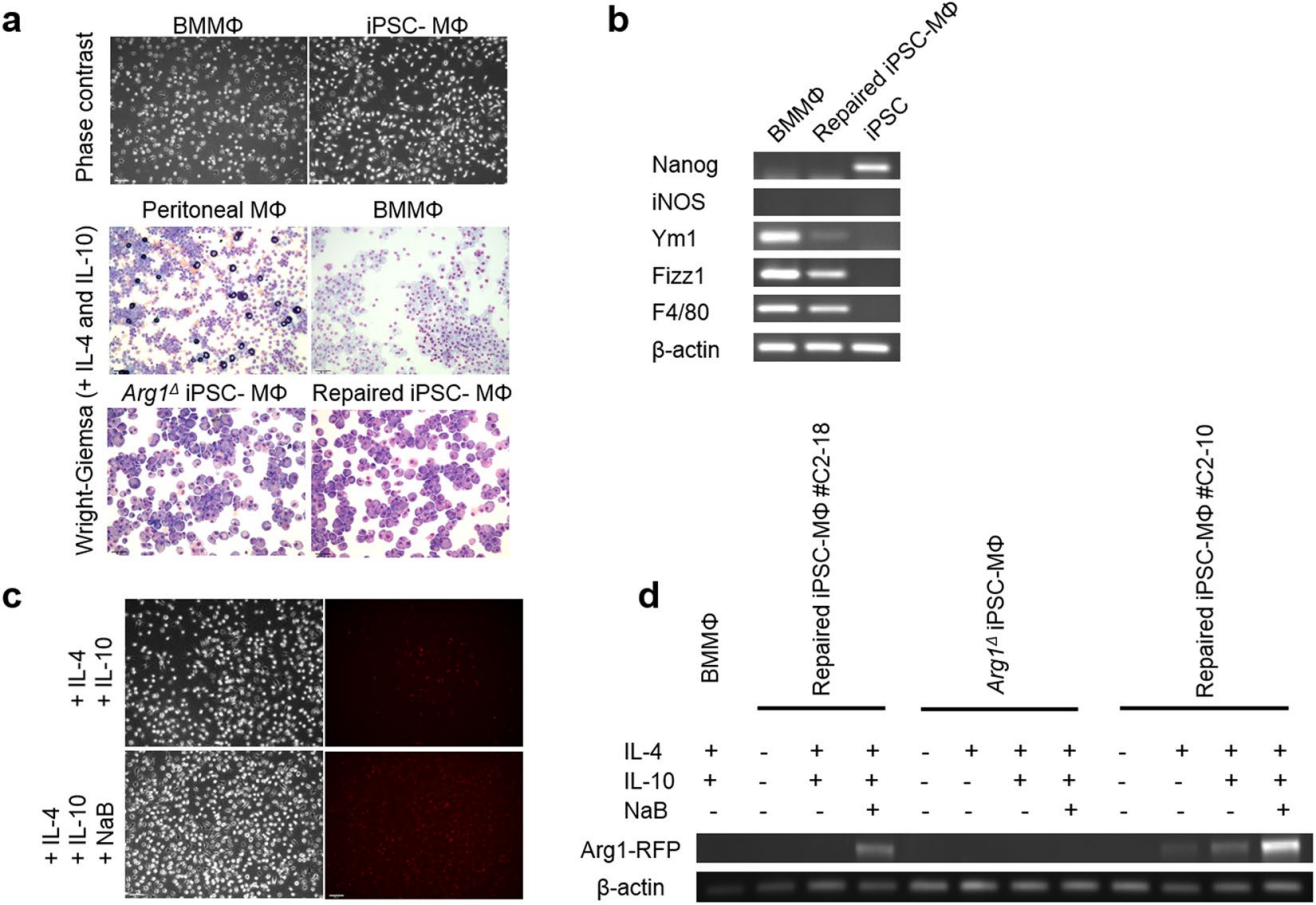
Characterization of iPSC-derived macrophages by assessing mRNA expression of macrophage phenotypic markers. (**a**) Phase contrast (upper panel) and cytocentrifuge preparations of macrophages for Wright–Giemsa staining (lower panels). The cells appear purplish and granular. 20x magnification. (**b**) RT-PCR analysis of macrophage marker genes in macrophages. Differentiated macrophages demonstrated M2-like characteristics. Expression of genes was normalized to β-actin. (**c**) RFP signal observed in the repaired iPSC-derived macrophages. (**d**) mRNA expression levels of Arg1-RFP in macrophages after treatment with IL-4, IL-10, sodium butyrate (NaB) as assessed by RT-PCR.

Given that iHLCs did not show the intended result with the fluorescent fusion protein, we opted to differentiate the repaired iPSCs to another cell type that strongly exhibits Arg1 expression in mice, namely macrophages of the M2 phenotype^31^. Macrophages derived from both repaired and non-edited iPSC lines analyzed at day 20 of differentiation showed similar morphology (bi-/multi-polar with round nucleus and abundant foamy cytoplasm when stained with Wright-Giemsa) to bone marrow-derived and peritoneal macrophages (Fig. 5a). RT-PCR analysis revealed an M2 phenotype of iPSC-derived macrophages (named iPSC-MΦ hereafter) upon stimulation of IL-4 and IL-10 with evidence for F4/80, Fizz1, Ym1 and no iNOS expression, with none of these markers present in iPSCs (Fig. 5b). Induction of arginase-1 was also observed at both the protein (Fig. 5c) and mRNA (Fig. 5d) levels. Importantly, macrophages derived from repaired iPSC lines, but not non-repaired cells, were able to express Arg1-RFP by fluorescence analysis (Fig. 5c) and by RT-PCR using primers within Arg1 and RFP domains (Fig. 5d). Gratifyingly, the expression of Arg1-RFP fusion was further upregulated upon sodium butyrate treatment (Fig. 5c,d). Therefore, precision editing of *Arg1* ^*∆*^ alleles with functional correction has occurred in differentiated macrophage populations, indicating that the overall strategy was successful.

## Discussion

The focus of this work relates to basic investigations into developing a gene repair strategy for a rare genetic disorder such as Arg1 deficiency, so as to halt progression of the disease. In this study, PMEFs obtained from our inducible Arg1-deficient mouse model were reprogrammed into iPSCs as a proof-of-concept system to exploit CRISPR/Cas9-mediated gene editing. The Arg1-deficient iPSCs exhibited typical embryonic stem cell-like characteristics and possess differentiation ability into three germ layers. Here, we used an all-in-one vector system expressing Cas9 and gRNA for our gene targeting approach. The functionality of the CRISPR/Cas9 system was validated using plasmid-mediated electroporation of iPSCs. Our results showed that this gene editing tool could induce precise sequence alterations within a defined locus of the *Arg1* gene. Sequence repair was mediated by a targeting vector carrying exons 7 and 8 cDNA-RFP fusion construct and selection cassette. We showed that a combination of CRISPR/Cas9 and *piggyBac* technology can achieve biallelic repair of multi-exon deletion in the *Arg1* gene without leaving any residual ectopic sequences at the site of correction. The positive-negative drug selection-based enrichment strategy enables efficient isolation of multiple correctly targeted and transgene-free repaired iPSCs.

For functional studies, the repaired iPSCs were first differentiated into hepatocyte-like cells to assess the restoration of *Arg1* gene expression. We successfully generated iHLCs that not only express hepatic markers, but also exhibited some normal hepatic functions including glycogen storage and metabolic capabilities, albeit weak, such as urea production. Unfortunately, the differentiated iHLCs did not show evidence of RFP fluorophore expression from the fusion protein. In our targeting vector, the RFP was designed to fuse immediately downstream of exon 8 to aid live-cell imaging. RFP expression of the fusion protein should be controlled and driven by the endogenous promoter of *Arg1*, thereby preventing possible overexpression-induced artefacts. In this regard, it is likely that the iHLCs were not fully mature in culture to the stage where the fusion protein could be expressed abundantly. This postulate was supported by the expression profile indicating that the iHLCs exhibited an immature phenotype (persistent expression of Afp and considerably low expression of the urea cycle enzymes) with minimal urea production compared to primary adult mouse hepatocytes (Fig. 4). To have a clearer assessment of the various experimental parameters relating to cell maturation status and fluorescent fusion expression, we turned to another cell type known to express Arg1 i.e murine macrophages of the M2 phenotype^31^. Thus, iPSCs were differentiated into macrophages and attained an appropriate degree of functional maturity, confirmed by the expression of F4/80, a marker preferentially expressed by mature macrophages^32^. Interestingly, we observed specific red fluorescent signal in the repaired iPSC-MΦ, which would indicate complete chromophore maturation. Undoubtedly, fluorescent fusion protein expression is likely to vary according to the cell type and degree of maturation.

There are several stages leading to the formation of an optically active chromophore within fluorescent proteins that must occur in a correct sequential manner. Problems can arise at each of the different steps involved, including translation, protein folding and maturation, oligomerization, photobleaching and degradation, with the time course of chromophore formation and protein degradation rates often more difficult to ascertain experimentally^33^. In general, fluorescent brightness is determined by a number of parameters, including cellular environment, expressing cell type, protein expression level and fusion protein partner^34, 35^. Previous studies have shown that fluorescence proteins mature at different rates depending on the type of cell in which they are being expressed^36^. Another explanation for the fusion protein that failed to express correctly in iHLCs may be due to chromosomal modification of histones. Indeed, addition of sodium butyrate to the culture medium enhanced the overall *Arg1*-*RFP* mRNA expression and fluorescence brightness at least in iPSC-MΦ. Given the role of butyrate in the modulation of genome expression by altering chromatin structure^37^, this might help in explaining the differentia1 effects on the efficiency of chromophore maturation in the cells. Whether this individual response to butyrate, a histone deacetylase inhibitor (HDACi)^38^, is related to histone modification normally occurring in this cell type is unknown. Previous investigations have demonstrated increased production of mature cystic fibrosis transmembrane conductance regulator (CFTR) upon butyrate stimulation^39, 40^. The mechanism is still not fully elucidated but is likely to involve the modulation of protein folding.

After we submitted this manuscript, Lee *et al*.^41^ reported a CRISPR/Cas9-based strategy utilizing exon 1 of the hypoxanthine-guanine phosphoribosyltransferase (HPRT) locus to genetically modify and restore arginase activity using hyperargininemic patient-derived cells. In line with our current study, their differentiated hepatocyte-like cells co-express Afp and Alb, indicating an immature phenotype as opposed to adult hepatocytes, which is also typically found in many differentiation cultures of mouse and human cells to date. The differences in arginase expression and urea production level in our differentiated iHLCs vs their system may be explained by the chosen HPRT knock-in locus, as well as the hepatic differentiation protocols used. Although they observed a high arginase RNA level and urea production in their corrected undifferentiated hiPSCs, likely due to the ubiquitously expressed *HPRT* gene, there was a significant decline in both ARG1 expression and functionality post-differentiation. The decrease is likely a consequence of their use of the elongation factor 1α (Ef1α) promoter in the targeting construct, which has been shown to decline in activity post-differentiation^42^, raising a concern about a potential further reduction in ARG1 expression and functionality after cellular transplantation. In contrast, our study took advantage of the *piggyBac* technology by employing a “pop in and out” repair strategy for the generation of seamlessly corrected cells under the influence of the endogenous *Arg1* promoter in the context of its native chromatin landscape.

Taken together, our work provides a proof-of-concept rationale for seamless gene editing of multi-exon deletion in *Arg1* using CRISPR/Cas9 in conjunction with *piggyBac* transposon technology. Moreover, we also provide important information about how fusion proteins may function differentially in different cell types. While we have successfully repaired Arg1 at the molecular genetic level, our current challenge is to differentiate iPSCs into mature hepatocytes at a comparable level to that of adult hepatocytes to verify functional correction. Our ongoing efforts include refining our current hepatic differentiation protocol, in particular the final step of differentiation to acquire a more mature phenotype. *In vivo* transplantations of both human and mouse immature iHLCs promote further maturation and long-term repopulation of hepatocytes^29, 43^. Thus, it is possible that the transplantation of gene-corrected iHLCs into our mouse model could enhance functional maturation. In addition, three-dimensional cultures or co-culture with non-parenchymal supportive cells before transplantation may be required to generate functional hepatocytes *in vitro* that can efficiently engraft into the mouse liver. Our initial studies using wild-type hepatocyte transplantation into inducible Arg1-deficient mice has not yet rescued animals from the lethal consequences of the disorder^12^. Successful gene editing and phenotypic corrections with gene-edited iPSC-derived cells still has obstacles to overcome before becoming a valuable tool to facilitate the development of liver-targeted cell therapies for urea cycle disorders.

## Methods

### Mice and cell source

The inducible Arg1-deficient mouse strain (herein referred to as *Arg1*-*Cre* mice) was derived from parental strains *Arg1* ^*flox*^ (JAX strain 008817, C57BL/6-*Arg1* ^*tm1Pmu*^/J) and CreER^T2^ (JAX strain 008463, B6.129-*Gt*(*ROSA*)*26Sor* ^*tm1*(*cre/ERT2*)*Tyj*^/J) and bred in-house as previously described^4^. All procedures were reviewed and approved by the Queen’s University Animal Care Committee (approval #Funk-2011-048-R1-A4) and conformed to the Guidelines of the Canadian Council on Animal Care. Primary mouse embryonic fibroblasts (PMEFs) were isolated from embryos of *Arg1*-*Cre* mating pairs 13.5 days post-coitum. Briefly, embryos were dissected and the head and the internal organs were removed. The remaining tissue was minced and digested overnight with 0.25% trypsin-EDTA (Hyclone) at 4 °C, followed by a 30 min incubation at 37 °C the next day to ensure complete tissue digestion. The obtained cells were seeded into gelatin-coated flasks and cultured in PMEF medium [high glucose Dulbecco’s modified Eagle’s medium (DMEM), 15% fetal bovine serum (FBS, Hyclone), 1% penicillin/streptomycin (Sigma)]. On day 1 and day 3 after seeding, PMEFs were treated with 0.5 µM 4-hydroxy-tamoxifen (OHT) (Sigma) to induce the deletion of exons 7 and 8 of *Arg1* (herein referred to as *Arg1* ^*Δ*^ PMEFs). PCR genotyping was carried out after day 5 of seeding to confirm Cre-excision using PCR with primer sets as follows: F1 *5*′-TGCGAGTTCATGACTAAGGTT-*3′*, R1 *5*′-AAAGCTCAGGTGAATCGG-*3′* and R2 *5*′-GCACTGTCTAAGCCCGAGAGTATC-*3′* ^4^.

To prepare feeder layers for culture of iPSCs, wildtype PMEFs were generated from individual mouse embryos (C57BL/6 background) and inactivated with 10 µg/ml of mitomycin C (Sigma) at passages 3–5 (P3-5). Puromycin-resistant, mitotically-inactivated PMEFs (iPMEFs) for drug selection experiments were a kind gift of Dr. Peter Greer (Queen’s University).

### Generation and characterization of *Arg1*^*Δ*^ mouse iPSCs

*Arg1* ^*Δ*^ mouse iPSCs were generated from *Arg1* ^*Δ*^ PMEFs using a polycystronic lentiviral construct expressing the canonical reprogramming factors Oct4, Klf4, Sox2 and c-Myc^25^ (STEMCCA Cre-Excisable Constitutive Polycistronic Lentivirus Reprogramming Kit, Millipore). All cell cultures were maintained at 37 °C/5% CO_2_. 3 × 10^4^ PMEFs (≤P3) were plated on a 6-well plate and cultured with PMEF medium supplemented with 5 µg/mL Polybrene (Millipore). The cells were infected with a multiplicity of infection (MOI) of 30 and the PMEF medium was refreshed every other day. Eight days after initiation of viral transduction, the cells were switched to mES medium [Knockout DMEM (Gibco), 15% ES-qualified FBS (Hyclone), 5% knockout serum replacement (KOSR, Gibco), 1% GlutaMax (Gibco), 1% penicillin/streptomycin, 0.1% β-mercaptoethanol (Gibco), 1,000 U/ml leukemia inhibitory factor (LIF, Gibco)] and refreshed every other day. Colonies were visible on day 14 and picked 4 days later. The colonies with good morphology were manually excised and expanded to 24-well plates containing iPMEFs for derivation of individual iPSC lines.

Alkaline Phosphatase Live Stain (Life Technologies) was used to check for pluripotency of the reprogrammed mouse iPSCs. For formation of embryoid bodies, iPSCs were cultured in ultra-low attachment dishes (Nunclon Sphera). For teratoma formation assay, 2 × 10^6^ undifferentiated mouse iPSCs were injected into the testicular capsules of NOD/SCID mice and grown for 8 weeks prior to sacrifice. Surgically-removed teratomas were fixed in 4% paraformaldehyde and embedded in paraffin. Sections were stained with hematoxylin and eosin for histologic evaluation of the three germ layers.

### Design and construction of CRISPR/Cas9 plasmids

The pX330 plasmid was obtained from Addgene (plasmid #42230)^20^. We used the online CRISPR Design Tool of the Zhang lab (http://crispr.mit.edu/) to identify suitable target sites in intron 6 upstream of the remnant LoxP site and assess the potential occurrence of off-target regions. To ensure targeting specificity, one unique target site with no homology elsewhere in the mouse genome was chosen. The *Arg1* gRNA target sequence, 5′-GCGCTGACAGGAATGTCAGG(AGG)-3′ was cloned into the BbsI site of the pX330 vector as described by Ran *et al*.^21^. The last three nucleotides (AGG) at the 5′-end is the PAM motif. The sequence of the Cas9:gRNA expression vector was confirmed by sequencing prior to experimental use.

### Functional evaluation of targeted DNA cleavage by Cas9:gRNA

*Arg1* ^*Δ*^ iPSCs (3 × 10^6^) were electroporated with 15 µg Cas9:gRNA plasmid DNA to induce double-stranded breaks (Bio-Rad Gene Pulser System: 250 V, 500 μF, 0.4 cm cuvettes). Genomic DNA from the electroporated cells was isolated two days later. The genomic region surrounding the gRNA target site was amplified with a high-fidelity polymerase (Phusion, NEB). Purified PCR products were subjected to a re-annealing process using a step gradient (95–25 °C over 30 min) to enable heteroduplex formation. For mismatch cleavage assays^44^, the annealed products were treated with Surveyor nuclease (Transgenomics) and resultant cleavage products were analyzed in 8% Tris-borate-EDTA polyacrylamide gels. The indel efficiency was quantified based on the relative band intensities measured using Quantity One Software (Bio-Rad). The indel percentage was calculated using the following formula: 100 × (1 − (1 − (b + c)/(a + b + c))^1/2^), wherein a is the intact band, b and c represent the Surveyor nuclease digestion products. For detection of targeted genome modification, PCR amplicons were subcloned into pCR 2.1-TOPO TA vector (Invitrogen) and individual colonies were subjected to sequence analysis.

A custom-designed repair targeting vector with *Arg1* homology arms, which consists of exons 7 and 8 cDNA fused to the coding sequence of monomeric RFP, a hybrid PGK-EM7 promoter, a positive-negative drug resistance cassette carrying a puromycin-thymidine kinase resistance gene, a self-cleaving 2A peptide (T2A), all flanked by the *piggyBac* transposon inverted terminal repeats (ITR) (Transposagen, Lexington, KY) was introduced into the cells together with the Cas9:gRNA plasmid to initiate HDR. The targeting vector was linearized with NotI prior to electroporation. L-755,507 (5 µM, Tocris BioScience) was also added to the cells to increase the efficiency of donor incorporation^27^. Puromycin treatment (1 µg/ml) was initiated four days after electroporation to identify cells that successfully integrated the targeting vector. The resulting colonies were picked on day 14 and maintained on puromycin-resistant iPMEFs. PCR genotyping was carried out for both left and right arms independently using primers as follows: left arm forward (LA-F) 5′-GTCTGCAGAGATTCGGAAGGTAAC-3′, reverse (LA-R) 5′-CTGACTAGGGGAGGAGTAGAAGGT-3′; right arm forward (RA-F) 5′-GGGACTGACTACCTTAAACCACCT-3′, reverse (RA-R) 5′-GGCTATTGAAGATTTAACATTTGG-3′. One of the two PCR primers was designed to anneal outside the region spanned by both homology arms to ensure on-target integration. LongAmp Taq DNA polymerase (NEB) was used for amplification of both arms. For the detection of random integrations of the targeting vector, the following primers were used to amplify both sides of the homology arm-plasmid junction individually: left arm forward (LRI-F) 5′-CTGCAAGGCGATTAAGTTGGGTAACG-3′, reverse (LRI-R) 5′-CACGATGTCTTTGGCAGATATGCAGG-3′; right arm forward (RRI-F) 5′-CCGTCAAGACTTTTCACATGCAGTC-3′, reverse (RRI-R) 5′-GCTCGTATGTTGTGTGGAATTGTGAG-3′. The selection cassette was removed by introducing *piggyBac* transposase (PBx) (Transposagen) into the puromycin-resistant cells. Four days after *piggyBac* excision, ganciclovir selection (2 µM, Cayman Chemical) was carried out to eliminate the cells that have residual vector expression. After a 2-week selection period, colonies were picked and expanded on gelatin-coated dishes without feeder cells. Genomic DNA was extracted and subjected to PCR-based screening of transposon-excised clones using primers as follows: PBx-F 5′-TCACAGGACTTACAGTGATC-3′; PBx-R 5′-CATGAACTCCTTGATGACG-3′ and PB3-P2-Forward 5′-GCGACGGATTCGCGCTATTTAGAAA-3′ (transposon-specific primer). PCR products resulting from the removal of the selection cassette were sequenced to confirm the presence of the TTAA sequence at the excised site^22^ and the intended genetic modification. For detection of transposon re-integration, the following primers were used: PB-F 5′-CTGCTGCAACTTACCTCCGGGATG-3′ and PB-R 5′-CCCCCAGAATAGAATGACAC-3′.

### Differentiation of iPSCs into hepatocyte-like cells *in vitro*

To confirm functional correction, the corrected cells adapted to feeder-free culture conditions were differentiated into hepatocyte-like cells (HLCs) using a modified stepwise protocol as previously described^29^. Mouse iPSCs were trypsinized into single cell suspensions and resuspended in advanced RPMI containing 10% FBS, 100 ng/ml activin A (R&D Systems) and 50 ng/ml Wnt3a (R&D Systems). One million cells per well were seeded into 12-well plates coated with 2% Matrigel (BD Biosciences) and incubated at 37 °C/5% CO_2_ for direct definitive endoderm (DE) induction without first undergoing embryoid body formation. FBS was reduced to 0.2% on the following day until day 5. On day 6–10, the medium was replaced with hepatic commitment medium [advanced RPMI, 2% FBS, 50 ng/ml BMP4 (PeproTech), 20 ng/ml FGF-2 (PeproTech). On day 11–15, the medium was replaced with advanced RPMI containing 2% FBS and 20 ng/ml HGF (PeproTech) to promote the expansion of early hepatic progenitor cells. To induce hepatocyte maturation, cells were cultured in advanced RPMI supplemented with 2% FBS, 20 ng/ml HGF, 20 ng/ml Oncostatin M (R&D Systems), 50 nM dexamethasone and 1x insulin-transferrin-selenium (Corning) for 10 days. The medium was changed every two days. Visualization of RFP signal was performed with a fluorescent microscope (Leica, DM IRB, Richmond Hill, ON) fitted with an excitation filter of 545 nm and an emission filter of 610 nm for mRFP.

### Isolation of primary hepatocytes

Primary hepatocytes were isolated from mice using a modified two-step collagenase perfusion system as described previously^4^.

### Periodic Acid Schiff (PAS) staining

Glycogen storage was assessed by Periodic Acid Schiff (PAS) staining. Cells were fixed 15 min in 4% paraformaldehyde, washed with PBS, permeabilized with 1% Triton-PBS and treated with Periodic Acid solution followed by Schiff solution as described by the manufacturer (Sigma Aldrich).

### Urea quantification

Urea production was measured as described previously^4^.

### Differentiation of mouse iPSCs into functional macrophages *in vitro*

Macrophage differentiation of iPSCs was performed as previously described^45^. After 20 days of differentiation, we examined the morphological and functional characteristics of these cells. TH2 cytokines, 10 ng/ml IL-4 and 10 ng/ml IL-10 (R&D System) were added into the culture for specific induction of arginase-1 in macrophages. Repaired iPSC-macrophages were compared to their non-repaired counterparts (parental cells) for evidence of phenotypic characterization.

### Preparation of primary macrophages

Unstimulated peritoneal cells were collected by lavage of wild-type adult C57BL/6 mice using ice-cold phosphate-buffered saline (PBS). Recovered cells were centrifuged, resuspended in RPMI 1640 + 10% FBS + 1% penicillin/streptomycin and cultured for 2 h at 37 °C/5% CO_2_ after which non-adherent cells were removed by washing. Purified macrophages were cultured for 24 hours before further manipulation.

Bone marrow-derived macrophages (BMMΦs) were obtained from wild-type adult C57BL/6 mice femurs using aseptic techniques. Briefly, marrow cores were flushed into sterile tubes using syringes fit with 23G needles and filled with flushing media [Iscove’s Modified Dulbecco’s Medium (IMDM), 2% FBS, 1% penicillin/streptomycin, 50 µM α-monothioglycerol (Sigma)]. Isolated cells were washed once in media, then cultured in macrophage media [IMDM, 15% FBS, 1% penicillin/streptomycin, 50 µM α-monothioglycerol, 30% L929 cell conditioned medium (as a source of macrophage colony stimulating factor to drive bone marrow cells towards a macrophage phenotype)] in 10 cm bacteriological grade Petri dishes. Non-adherent cells were collected at day 4 and replated in macrophage media at a density of 6 × 10^6^ cells/well in 6-well plates. On day 7, resultant cultures were either treated with 10 ng/mL IL-4 (R&D Systems) and 10 ng/mL IL-10 (R&D Systems) to stimulate M2 conditions^31^ or received media alone (M0 condition). Cells were harvested 24 h post-stimulation for total RNA extractions.

### Cytospin centrifugation and staining

Cells were placed on slides by centrifugation at 700 rpm for 5 min (CytoSpin 3 Cytocentrifuge, Shandon). Slides were air-dried prior to staining. For morphological analysis, slides were stained with modified Wright–Giemsa stain (Sigma-Aldrich) according to the manufacturer’s protocol.

### Gene expression analysis

RNA was extracted from cells using GeneJET RNA Purification Kit (ThermoFisher) and treated with DNAse I (1 µg/µl, Invitrogen) as per the manufacturer’s instructions. cDNA was generated using an iScript cDNA synthesis kit (Bio-Rad). For RT-PCR, primers (Integrated DNA Technologies) were designed to span exon-exon boundaries to avoid the amplification of genomic DNA. The primer sequences of the genes studied are listed in Table 1. Expression of individual transcripts was normalized to β-actin expression. Quantitative PCR was performed using a thermal cycler (Applied Biosystems Model 7500) with SYBR Green PCR master mix (BioRad). Melting curves were performed on completion of the cycles to ensure absence of nonspecific products. Relative gene expression was calculated using the comparative threshold method (2^−ΔΔCT^) and is presented as fold-change of transcripts for target genes normalized to the expression of housekeeping gene 18S rRNA. Gene expression values in undifferentiated iPSCs were set to 1.

**Table 1:** Primer sequences for RT-PCR analysis.

Gene	Accession No.		Sequence (5′–>3′)
*Nanog*	NM_028016	Forward	AGGACAGGTTTCAGAAGCAGA
		Reverse	CCATTGCTAGTCTTCAACCACTG
*α*-*fetoprotein*	NM_007423	Forward	CACTGCTGCAACTCTTCGTA
		Reverse	CTTTGGACCCTCTTCTGTGA
*Albumin*	NM_009654	Forward	GACAAGGAAAGCTGCCTGAC
		Reverse	TTCTGCAAAGTCAGCATTGG
*Arg1* (*Exon 7-8*)	NM_007482	Forward	ACAAGACAGGGCTCCTTTCAG
		Reverse	TGAGTTCCGAAGCAAGCCAA
*β*-*actin*	NM_007393	Forward	GACCTCTATGCCAACACAGT
		Reverse	AGTACTTGCGCTCAGGAGGA
*iNOS*	NM_010927	Forward	CACCAAGCTGAACTTGAGCG
		Reverse	CGTGGCTTTGGGCTCCTC
*Ym1*	NM_009892	Forward	CAAGTTGAAGGCTCAGTGGCTC
		Reverse	CAAATCATTGTGTAAAGCTCCTCTC
*Fizz1*	NM_020509	Forward	CTGCCCTGCTGGGATGACT
		Reverse	CATCATATCAAAGCTGGGTTCTCC
*F4/80*	NM_010130	Forward	CTTTGGCTATGGGCTTCCAGTC
		Reverse	CAAGGAGGACAGAGTTTATCGTG
*mRFP*	AF506027	Reverse	GCTTGATGTCGGTCTTGTAGG
*Cps*	NM_001080809	Forward	TCGGGATAAGGGTACCATGC
		Reverse	GCTTAACTAGCAGGCGGATG
*Otc*	NM_008769	Forward	AGGGTCACACTTCTGTGGTTC
		Reverse	CAGAGAGCCATAGCATGTACTG
*Ass1*	NM_007494	Forward	ACACCTCCTGCATCCTCGT
		Reverse	GCTCACATCCTCAATGAACACCT
*Asl*	NM_133768	Forward	CTATGACCGGCATCTGTGGAA
		Reverse	AGCAACCTTGTCCAACCCTTG
18S rRNA	NR_003278	Forward	CTTAGAGGGACAAGTGGCG
		Reverse	ACGCTGAGCCAGTCAGTGTA

*iNOS*, inducible nitric oxide synthase; *Ym1*, chitinase-like 3 (*Chil3*); *Fizz1*, resistin-like alpha (*Retnla*); *Cps*, carbamoyl-phosphate synthetase 1; *Otc*, ornithine transcarbamylase; *Ass1*, argininosuccinate synthetase 1; *Asl*, argininosuccinate lyase.

### Statistical analysis

All experiments were performed at least in triplicates. Results are expressed as mean ± standard deviation (SD). Statistical analysis was performed using GraphPad Prism 6 (GraphPad Software, San Diego, California, USA). Means were compared using the two-tailed Student’s t-test. *P* values of < 0.05 were considered statistically significant.
